# Residual renal function in chronic dialysis is not associated with reduced erythropoietin-stimulating agent dose requirements: a cross-sectional study

**DOI:** 10.1186/s12882-017-0752-x

**Published:** 2017-11-25

**Authors:** Elizabeth Helene Louw, Mogamat-Yazied Chothia

**Affiliations:** Divisions of General Medicine and Nephrology, Department of Medicine, Faculty of Medicine and Health Sciences, Stellenbosch University and Tygerberg Academic Hospital, Cape Town, 7505 South Africa

**Keywords:** Residual renal function, Erythropoietin resistance index, Chronic dialysis, Erythropoietin stimulating agent dose requirements

## Abstract

**Background:**

Anaemia is a very common problem in patients with end-stage kidney disease (ESKD) and the use of erythropoietin-stimulating agents (ESA) has revolutionised its treatment. Residual renal function (RRF) is associated with a reduction in ESA resistance and mortality in chronic dialysis. The primary aim was to establish whether RRF has an association with ESA dose requirements in ESKD patients receiving chronic dialysis.

**Methods:**

A single center, cross-sectional study involving 100 chronic dialysis patients was conducted from December 2015 to May 2016. Participants were divided into two groups depending on presence of RRF, which was defined as a 24-h urine sample volume of ≥ 100 ml. Erythropoietin resistance index [ERI = total weekly ESA dose (IU)/weight (kg)/haemoglobin concentration (g/dL] was used as a measure of ESA dose requirements.

**Results:**

There was no difference in ERI between those with RRF as compared to those without (9.5 versus 11.0, respectively; *P* = 0.45). Also, ERI did not differ between those receiving haemodialysis as compared with peritoneal dialysis (10.8 versus 10.2, respectively; *P* = 0.84) or in those using renin-angiotensin system (RAS) blockers as compared with no RAS blocker use (11.6 versus 9.2, respectively; *P* = 0.10). Lower ERI was evident for those with cystic kidney disease as compared to those with other causes of ESKD (6.9 versus 16.5, respectively; *P* = 0.32) although this did not reach statistical significance. Higher ERI was found in those with evidence of systemic inflammation as compared to those without (16.5 versus 9.5, respectively; *P* = 0.003).

**Conclusions:**

There was no association between RRF and ESA dose requirements, irrespective of dialysis modality, RAS blocker use, primary renal disease or hyperparathyroidism.

## Background

Anaemia is a very common problem in end-stage kidney disease (ESKD) and has been reported to occur in up to 75% of patients at presentation [1]. The treatment of anaemia with erythropoietin-stimulating agents (ESA) in ESKD has revolutionised its treatment, but its use has been tempered by higher risks of cardiovascular morbidity and mortality [2]. This has resulted in a more judicious prescription of ESA in chronic dialysis patients with more conservative haemoglobin (Hb) target ranges. The focus of treatment of anaemia has shifted from reducing cardiovascular event rates to improving quality of life.

A recent meta-analysis found no difference in Hb concentrations between haemodialysis (HD) and peritoneal dialysis (PD) patients [3]; however, treatment response to ESA may vary depending on dialysis modality. It has been reported that PD patients tend to have lower ESA dose requirements than their HD counterparts [4, 5]. Possible explanations for this include less frequent phlebotomy, subcutaneous administration of ESA that is associated with up to a third saving in dose and finally the preservation of residual renal function (RRF) [6]. The most common factors associated with increased dose requirements in chronic dialysis patients are iron deficiency and chronic inflammation. Other causes include malnutrition, hyperparathyroidism, poor vascular access, older age, dialysis vintage and use of renin-angiotensin-system (RAS) blockers [7, 8].

A very important factor associated with reduced ESA dose requirements is RRF. There is no universally accepted definition for RRF, although most studies make use of a urine volume greater than 100 ml to 250 ml per day [9, 10]. The CANUSA study reported that for every 250 ml of urine output per day, mortality was reduced by 36% [9]. Other studies reported that the risk of death was reduced by 11–23% for each 1 ml/min/1.73m^2^ of RRF [10, 11]. It has been found that RRF is better preserved in PD than HD [12]. This may be due to improved haemodynamic stability during ultrafiltration in PD as compared to HD. Therefore, RRF may partially explain why PD patients have reduced ESA dose requirements as well as less ESA resistance.

Erythropoietin resistance index (ERI), which is defined as the weekly dose of ESA divided by patient weight and corrected for the Hb concentration, is a measure of a patient’s response to ESA. It has become routine for studies to report ERI as a measure of ESA dose requirements. A recent study found that a strong linear relationship exists between ERI and weight-adjusted ESA dose using a universal formula [13]. However, it is important to take cognisance of the timing of the ESA initiation and the value of the accompanying Hb concentration when calculating the ERI. At the time of initiation, a higher ERI measurement may be evident as lower Hb concentrations may be present. As the Hb concentration increases in response to the ESA, the ERI will decrease [14]. Once target Hb concentrations are achieved, the ESA dose is unlikely to change and the ERI calculation will remain relatively constant. Therefore, an ERI which is high at the time of ESA initiation may wrongly be interpreted as ESA resistance. ERI should only be used in patients established on relatively constant ESA doses to avoid misinterpretation and inappropriate increases in dose. It has been reported that the ERI is significantly lower in patients with RRF when compared to those with anuria [15, 16].

The primary aim of this study was to establish whether RRF in chronic dialysis patients was associated with reduced ESA dose requirements using ERI as a measure of response. Secondary outcomes included comparisons of ERI between dialysis modality, route of ESA administration, primary renal disease and RAS blocker use.

## Methods

### Study design, setting and participants

**Fig. 1:**
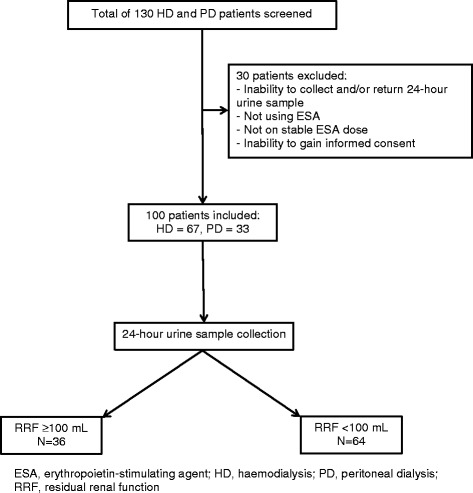
Flow diagram of patients screened and included in the final analysis

A cross-sectional study involving 100 chronic HD and PD patients was undertaken at Tygerberg Academic Hospital and its associated satellite dialysis units in the Western Cape province of South Africa, during the period of December 2015 to May 2016. All chronic dialysis patients ≥ 18 years of age, with the ability to give informed consent and on stable ESA treatment for at least 3 months were included. Patients were excluded if they were receiving dialysis for acute kidney injury or delayed graft function, actively bleeding, current infection or there was an inability to collect a 24-h urine sample (Fig. 1). All patients included were free of primary haematological diseases.

Data regarding demographics, primary renal disease, co-morbid diseases, dialysis modality and vintage, dry weight and drug prescription were collected from clinical records. Laboratory data collected included Hb, albumin, ferritin, transferrin saturation, C-reactive protein (CRP) and parathyroid hormone (PTH). RRF was measured using a urine sample collected over 24-h. In HD patients, it was measured during the longest inter-dialytic period and in PD patients during any 24-h period.

The patients were then divided into two groups depending on presence or absence of RRF, defined as the production of at least 100 ml of urine in 24 h (Fig. 1). ERI for each patient was calculated as the current weekly erythropoietin dose per kilogram of body weight (IU/kg/week) divided by the Hb concentration (g/dL). All the patients received ESA subcutaneously. Laboratory evidence of systemic inflammation was defined as the presence of all the following: a low serum albumin (< 35 g/L), raised serum CRP (> 10 mg/L) and raised serum ferritin (> 150 ng/mL). Hyperparathyroidism was classified according to the Kidney Disease Improving Global Outcomes (KDIGO) clinical practice guideline recommendations for the management of chronic kidney disease-mineral and bone disorder (CKD-MBD). Those with a PTH value of 2–9 times the upper limit of normal for the assay were regarded as being in the target range (17.0–76.5 pmol/L). Those with PTH < 17.0 pmol/L or >76.5 pmol/L were regarded as being over-suppressed or having hyperparathyroidism, respectively.

### Statistical analysis

Means ± standard deviations were used to summarize data with a normal distribution and medians and inter-quartile ranges (IQR) for data that did not have a normal distribution. Histograms, bar graphs and box-and-whisker plots were used where appropriate. Chi-squared and Fisher’s exact tests were used to compare categorical data. Both unadjusted and adjusted analysis with multi-linear regression for age, sex and race were used for dialysis modality, route of ESA administration, PTH status, RAS blocker use and primary renal disease. Where continuous variables had a normal distribution, t-tests were used to compare means, and if not normally distributed were analysed using Mann-Whitney-U tests. A significant *P*-value was set at *P* < 0.05. SPSS version 24 was used for data analysis.

## Results

A total of 100 patients were included in the final analysis. The mean for age was 41 ± 10.5 years and 57% were female. Most of the patients were of mixed ancestry (65%). The most common primary kidney disease was ESKD (cause unknown) (66%), followed by renal vascular disease (14%). Two-thirds (67%) of the patients were receiving chronic HD as their dialysis modality while the rest were receiving chronic ambulatory PD.

A total of 36 patients had RRF. It was more common in PD patients than in those receiving HD as their dialysis modality (58.3% versus 41.7%, respectively; *P* < 0.01). Patients with RRF were younger (37.4 versus 43.0 years old; *P* = 0.01), and had a shorter dialysis vintage (34 versus 64 months; *P* < 0.01). More patients with RRF were on diuretics (75.0% versus 31.3%, *P* < 0.01) and phosphate binders (94.4% versus 73.4%, *P* = 0.01). There was a trend toward less RAS blocker use in those with RRF as compared to those without (38.9% versus 59.4%, respectively; *P* = 0.05). There were no statistical significant differences in any of the laboratory serum measurements (Table 1).

**Table 1:** Comparisons of clinical baseline characteristics in those with and without RRF

Parameter	RRF present		RRF absent		*P*-value
Number of patients	36		64		–
Age in years, mean ± SD	37.4	±10.7	43	±10.2	0.01
Sex, n (%)					
Male	15	(41.7)	28	(43.8)	0.84
Female	21	(58.3)	36	(56.3)	
Race, n (%)					
Mixed ancestry	23	(63.9)	42	(65.6)	0.15
Caucasian	6	(16.7)	16	(25.0)	
Black	5	(13.9)	6	(9.4)	
Indian	2	(5.6)	0	(0)	
Underlying kidney disease, n (%)					
ESKD (cause unknown)	23	(63.9)	43	(67.2)	0.78
Renal vascular disease	6	(16.7)	8	(12.5)	
Cystic kidney disease	1	(2.8)	3	(4.7)	
Autoimmune disease	2	(5.6)	2	(3.1)	
Other and unknown	4	(11.1)	8	(12.5)	
Mode of dialysis, n (%)					
HD	15	(41.7)	52	(81.3)	<0.01
PD	21	(58.3)	12	(18.8)	
Dialysis vintage (months)	34	(10.5–53)	64	(41–125)	<0.01
ESA dose (IU per week)	6000	(5000–6000)	6000	(3000–9000)	0.46
Route of ESA administration, n (%)					
SC	36	(100)	64	(100)	–
Chronic medication, n (%)					
RAS blocker	14	(38.9)	38	(59.4)	0.05
Diuretic	27	(75.0)	20	(31.3)	<0.01
Phosphate binder	34	(94.4)	47	(73.4)	0.01
Vitamin D	9	(25.0)	27	(37.5)	0.08
Iron therapy	35	(97.2)	63	(98.4)	0.46
Oral	31	(86.1)	50	(78.1)	
IV	4	(11.1)	13	(20.3)	
Laboratory parameters					
Haemoglobin (g/dL)	10.1	(9.2–11.1)	10.1	(8.8–10.8)	0.54
Albumin (g/L)	39	(34–40)	37	(33–40)	0.28
Ferritin (ng/mL)	545.0	(299–759.5)	469.0	(237.5–781.5)	0.99
Transferrin saturation (%)	22	(15–30)	23	(16–29)	0.49
CRP (mg/L)	15	(4–29)	20	(4–45)	0.48
PTH (pmol/L)	41.5	(18.6–73.2)	50.2	(17.6–117.4)	0.70
Systemic inflammation, n (%)	3	(8.3)	11	(17.1)	0.21

Values expressed in a range in parentheses refer to interquartile ranges; single values refer to percentage of the group population. *RRF* residual renal function; *SD* standard deviation; *ESKD* end-stage kidney disease; *HD* haemodialysis; *PD* peritoneal dialysis; *ESA* erythropoietin-stimulating agent; *IU* international units; *SC* subcutaneous; *RAS* renin angiotensin system; *CRP* C-reactive protein; *PTH* parathyroid hormone. Systemic inflammation was defined as serum ferritin >150 ng/mL and serum CRP >10 mg/L and serum albumin <35 g/L

**Fig. 2:**
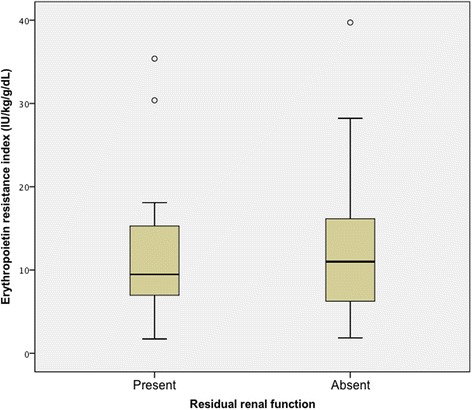
Primary outcome: Erythropoietin resistance index and residual renal function

**Fig. 3:**
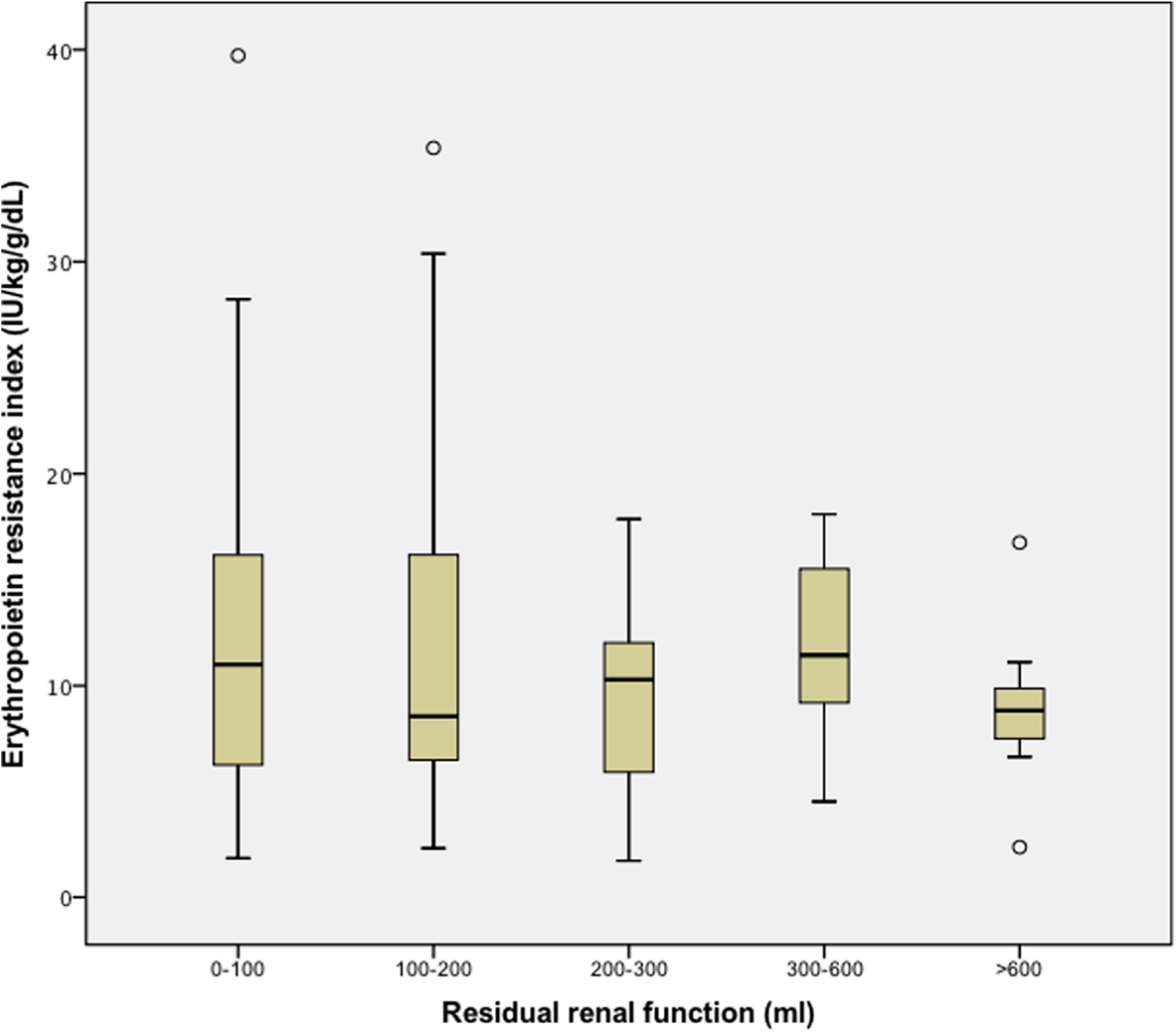
A comparison of quartiles of residual renal function and its association with erythropoietin resistance index

With respect to our primary outcome, ERI did not differ in those with or without RRF (9.5 [IQR 7.0–15.3] versus 11.0 [IQR 6.3–16.2], respectively; *P* = 0.45). The median dose of ESA was identical in each group (6000 IU per week). When dividing RRF into quartiles, there were no statistical differences in ERI between those with higher volumes of RRF as compared to those with lower volumes or no RRF (*P* = 0.82) (Figs. 2 and 3). Also, a statistically significant association was not identified between ERI and RRF volume using linear regression analysis (*P* = 0.27).

ERI did not differ by dialysis modality (10.8 [IQR 8.5–14.3] in PD versus 10.2 [IQR 5.9–16.0] in HD, *P* = 0.84). Lower ERI were evident for those with cystic kidney disease as compared to those with other causes of ESKD (6.9 [IQR 3.9–13.2] versus 16.5 [IQR 12.0–17.9], respectively; *P* = 0.43) but it did not reach statistical significance. There was no statistically significant difference in ERI in those with or without RAS blocker use (11.6 [IQR 7.9–16.7] versus 9.2 [IQR 6.1–15.2], respectively; *P* = 0.10).

A total of 14 patients fulfilled our criteria for systemic inflammation. Of these, 11 (78.6%) had no RRF and had higher ERI when compared to those without systemic inflammation (16.5 [IQR 11.0–18.7] versus 9.5 [IQR 6.0–14.9], respectively; *P* = 0.003). A statistically significant association between ERI and systemic inflammation persisted after adjusting for dialysis vintage using multiple linear regression, *P* = 0.049). When comparing ERI with PTH <17.0 pmol/L (over-suppressed), 17.0–76.5 pmol/L (target range) and >76.5 pmol/L (hyperparathyroidism), ERI tended to be higher in the over suppressed group but there were no statistical significant differences (Tables 2 and 3).

**Table 2:** Primary and secondary outcomes

Parameter	Number of patients	Median ERI	IQR	*P*-value
Primary Outcome				
Residual renal function				
Present	36	9.5	(7.0–15.3)	0.45
Absent	64	11.0	(6.3–16.2)	
Secondary Outcomes				
Dialysis modality				
HD	67	10.8	(8.5–14.3)	0.84
PD	33	10.2	(5.9–16.0)	
RAS blocker use	52	11.6	(7.9–16.7)	
ACE inhibitor	43	11.8	(8.1–16.8)	0.10
ARB	9	11.0	(6.0–13.7)	
No RAS blocker use	48	9.2	(6.1–15.2)	
PTH category (pmol/L)				
< 17	19	13.5	(6.5–16.8)	0.73
17–76.5	34	9.6	(6.2–14.9)	
> 76.5	28	9.1	(6.8–15.3)	
Systemic inflammation				
Present	14	16.5	(11.0–18.7)	<0.01
Absent	86	9.5	(6.0–14.9)	
Underlying kidney disease				
ESKD (cause unknown)	66	10.8	(7.8–15.4)	0.32
Renal vascular disease	14	8.4	(5.2–14.3)	
Cystic kidney disease	4	6.9	(3.9–13.2)	
Autoimmune disease	4	16.5	(12.0–17.9)	
Other and unknown	12	9.1	(6.4–15.5)	

*ERI* erythropoietin resistance index; *IQR* interquartile range; *HD* haemodialysis; *PD* peritoneal dialysis; *ACE* angiotensin-converting enzyme; *RAS* renin angiotensin system; *PTH* parathyroid hormone; *ESKD* end-stage kidney disease. ‘Other’ refers to reflux nephropathy, single kidney, renal cortical necrosis, pre-eclampsia, drug overdose and unknown aetiology. Systemic inflammation was defined as serum ferritin >150 ng/mL and serum CRP >10 mg/L and serum albumin <35 g/L

**Table 3:** Comparisons of clinical parameters between dialysis modalities

Parameter	Haemodialysis		Peritoneal dialysis		*P*-value
Number of patients	67	–	33	–	–
Residual renal function, n (%)					
Present	15	(22.4)	21	(63.6)	<0.01
Absent	52	(77.6)	12	(36.4)	
Dialysis vintage (months)	70	(41–125)	14.5	(10–47)	<0.01
Haemoglobin (g/dL)	10.1	(9.3–11)	9.5	(7.9–10.8)	0.05
ESA dose per week (IU)	6000	(6000–10,000)	6000	(6000–6000)	0.16
ERI	10.79	(5.93–16.02)	10.20	(8.06–14.25)	0.84

Values expressed in a range in parentheses refer to interquartile ranges; single values refer to percentage of the total population. *ESA* erythropoietin-stimulating agent; *ERI* erythropoietin resistance index

## Discussion

In our study, we found no association between RRF and ESA dose requirements as measured using ERI. This finding contrasts with some larger studies that identified less ESA requirements in those patients with preserved RRF [15, 16]. These studies were done mainly in HD patients. Another study that was conducted in PD patients reported no influence of RRF on ERI [17]. A recent, large observational study reported that ERI was higher for patients on HD as compared to those on PD [5]. It is unclear whether patients included were on stable ESA doses and what the effect RRF may have had. However, the authors mention that more frequent phlebotomy and the intravenous administration of ESA might have elevated the ESA dose requirements in the HD group. In contrast, all our participants received ESA by the subcutaneous route and were on stable ESA doses for at least 3 months prior to recruitment therefore limiting the effect that these two potential confounding variables may have had on ERI.

Our study population was younger and mainly of mixed ancestry when compared to other studies [18, 19]. Most were receiving HD with fewer PD patients included due to logistical issues related to the collection and/or the return of 24-h urine samples and an inability to obtain informed consent. RRF was mostly present in our PD patients who also had the shortest dialysis vintage. These findings reflect our PD-first policy.

We found a strong association between higher ERI and laboratory evidence of systemic inflammation. Others have reported similar findings [5, 17]. None of our patients were iron deficient or had an obvious infection. This systemic inflammation could possibly be due to occult infection or dialysis-related factors. We have previously reported a strong association between occult periodontal disease and systemic inflammation in our chronic dialysis population [20]. A high ERI may therefore be a marker of systemic inflammation and clinicians should be cautious when deciding to increase the ESA dose, as this class of drugs has been associated with increased risk of cardiovascular events.

We found no association between ERI and dialysis modality, RAS blocker use, the underlying primary renal disease or hyperparathyroidism. A recent meta-analysis also reported no difference relative to dialysis modality [3], although some individual studies have reported differences [4, 5]. There was a trend toward less RRF in those with RAS blocker use. Although there have been studies suggesting that RAS blocker use may preserve RRF, others have found no benefit [21, 22]. It may be that better volume control in those with RRF resulted in less prescription of antihypertensive drugs including RAS blockers. RAS blockers had no significant effect on ERI. The influence of RAS blockers on ESA resistance remains controversial. Various pathogenic mechanisms have been implicated [23, 24]; however, observational studies have reported mixed results [25–27]. Nonetheless, a dose-response relationship does exist [28] and therefore when other common causes for ESA resistance have not been identified, reducing or stopping RAS blockers is a reasonable next step.

The lower ERI in polycystic kidney disease patients is expected as it is thought that interstitial cells adjacent to the walls of the proximal-type cysts can produce erythropoietin, resulting in higher Hb concentrations and therefore lower ESA requirements [29]. Our patients with autoimmune disease tended to have higher ERI that may be related to systemic inflammation.

Hyperparathyroidism is frequently listed as a cause for ESA resistance; however, we did not identify any association with PTH level. In fact, our patients classified as having secondary hyperparathyroidism tended to have a lower ERI. It is thought that the high-turnover bone disease from secondary hyperparathyroidism may cause bone marrow fibrosis. This has not been found in animal models of secondary hyperparathyroidism [30] and it therefore seems increasingly unlikely that hyperparathyroidism contributes to ESA resistance.

### Strengths and limitations

Our study has some limitations. This was a relatively small, single center study, with fewer PD patients. The inclusion of 24-h urine sample creatinine clearance and/or β2-microglobulin may have better characterised RRF. However, the interpretation of our ERI measurements is more reliable because of the uniform route of ESA administration and the inclusion of only those on relatively stable ESA doses.

## Conclusions

In summary, we found no association between RRF and ESA dose requirements as measured using ERI, irrespective of dialysis modality, RAS blocker use, primary renal disease or hyperparathyroidism. However, the presence of systemic inflammation had a significant effect on ERI. Therefore, in those with high ESA dose requirements, an active search for a source of inflammation should be conducted.

## Abbreviations

CANUSA: Canada-USA Peritoneal Dialysis Study
CKD-MBD: Chronic kidney disease-mineral and bone disorder
CRP: C-reactive protein
ERI: Erythropoietin resistance index
ESA: Erythropoietin-stimulating agents
ESKD: End-stage kidney disease
Hb: Haemoglobin
HD: Haemodialysis
IQR: Interquartile range
KDIGO: Kidney Disease Improving Global Outcomes
PD: Peritoneal dialysis
PTH: Parathyroid hormone
RAS: Renin-angiotensin-system
RRF: Residual renal function
